# The need for continued monitoring of antibiotic resistance patterns in clinical isolates of *Staphylococcus aureus *from London and Malta

**DOI:** 10.1186/1476-0711-9-20

**Published:** 2010-07-21

**Authors:** Simon WJ Gould, Paul Cuschieri, Jess Rollason, Anthony C Hilton, Sue Easmon, Mark D Fielder

**Affiliations:** 1 School of Life Sciences, Kingston University, Kingston-upon Thames, UK, KT1 2EE; 2 Department of Microbiology, St. Luke's Hospital, Malta; 3 School of Life and Health and Life Sciences, University of Aston, Birmingham UK, B4 7ET

## Abstract

**Background:**

Antibiotic resistance is an increasing problem in isolates of *Staphylococcus aureus (S. aureus) *worldwide. In 2001 The National Health Service in the UK introduced a mandatory bacteraemia surveillance scheme for the reporting of *S. aureus *and methicillin-resistant *S. aureus *(MRSA). This surveillance initiative reports on the percentage of isolates that are methicillin resistant. However, resistance to other antibiotics is not currently reported and therefore the scale of emerging resistance is currently unclear in the UK. In this study, multiple antibiotic resistance (MAR) profiles against fourteen antimicrobial drugs were investigated for 705 isolates of *S. aureus *collected from two European study sites in the UK (London) and Malta.

**Results:**

All isolates were susceptible to linezolid, teicoplanin and vancomycin. Multiple antibiotic resistance profiles from both countries were determined, a total of forty-two and forty-five profiles were seen in the UK cohort (MRSA and MSSA respectively) and comparatively, sixty-two and fifty-two profiles were shown in the Maltese group. The largest MAR profile contained six antibiotics (penicillin G, methicillin, erythromycin, ciprofloxacin, clindamycin and clarithromycin) and was observed in the MRSA isolates in both the UK and Maltese cohorts.

**Conclusion:**

The data presented here suggests that the monitoring of changing resistance profiles locally in maintaining treatment efficacy to resistant pathogens.

## Background

*Staphylococcus aureus *and particularly methicillin-resistant *S. aureus *(MRSA) have become a major problem in hospital acquired infections (HAIs) worldwide. The World Health Organisation (WHO) recognizes that antibiotic resistance is one of the major threats facing the world in the future [1].

Between 1991 and 2000 the Department of Health (DoH) in the UK reported that bacteraemia cases caused by MRSA in the UK rose from 2% to 40% [2]. In April 2001 the National Health Service in the UK introduced a mandatory bacteraemia surveillance scheme for *S. aureus*. Current levels of *S. aureus *bacteraemia are reported to be between 9,000-10,000 cases per annum in the UK of which 38-40% of cases are attributed to MRSA[3,4]. However, these reports only state the incidence of methicillin resistance and do not include the occurrence of resistance to other clinically important antibiotics. As both methicillin-sensitive *S. aureus *(MSSA) and MRSA are resistant to multiple antibiotics, full multiple antibiotic resistance (MAR) profiles are required to aid successful therapy and maintain an understanding of the ever changing dynamics of antimicrobial resistance.

The aim of this study was to carry out a comparative assessment of MAR profiles in clinical isolates of *S. aureus*, collected from the three London based hospitals (LBH) and the main general hospital in Malta. The latest European Antimicrobial Resistance Surveillance System (EARSS) annual report [5] showed the proportion of MRSA bacteraemia across Europe between 2000 to 2008. The report demonstrated the that Malta has one of the highest proportion of MRSA bacteraemia in Europe, between this time the levels of MRSA ranged from 35 to 55%, with the highest level recorded in 2006 at 66%. The UK had the fifth highest level of MRSA in Europe over the same time period ranging from 31-34%, with the highest level recorded in 2001 at 44% [5]. Both these countries have reported sustained levels of MRSA bacteraemia at approximately 40% since 2001 and therefore represent counties where MRSA is considered an important problem.

## Materials and Methods

### Bacterial strains

Clinical isolates of MRSA and MSSA were collected from three LBH; Kingston Hospital, Surrey (a general hospital), The Royal Brompton Hospital (a specialist respiratory hospital), London; and The Royal Marsden Hospital, London (a specialist cancer hospital) and from St. Luke's Hospital, Malta (main general hospital in Malta serving a population of approximately 400,000 people). Participating hospitals were selected based upon having different specialties and so serving specific patient groups or alternatively a general hospital with a broad cohort of patients. A total of 378 isolates were collected from the LBH (260 MRSA and 118 MSSA) and 309 isolates were collected from the Maltese hospital (216 MRSA and 93 MSSA). All LBH *S. aureus *isolates were collected over an 18 month period (07/2002-12/2004) and the Maltese isolates were collected over an 8 month period (02/2003-10/2003). All bacterial isolates originated from swab samples taken from patients admitted to the study hospitals. Duplicate isolates from patient samples were excluded from the study. No further individual patient data was made available.

All isolates were also transferred to 1 ml aliquot of Brain-heart infusion broth (Oxoid Ltd), containing 15% glycerol (Sigma Ltd) and frozen at -80°C. The following *S. aureus *strains were used as controls during the experiment: MRSA NCTC 12493 (Control 1) and Oxford *S. aureus *NCTC06571 (Control 2), a strain sensitive to all antibiotics in the test panel.

### Isolates confirmation

Isolate identification was primarily determine in respective hospital. Identification of isolates was reconfirmed in the author's laboratory using a standard method (Gram staining and coagulase production using staphylase test (Oxoid, UK).

### Antibiogram testing

The antibiotic panel consisted of 14 different antimicrobials. Thirteen were tested using standard operating procedures defined by the BSAC Version 3 2004 [6]. In addition, vancomycin sensitivity was assayed using a previously published protocol [7]. The following antibiotics were tested using BSAC methodology: 30 μg amikacin, 10 μg chloramphenicol, 1 μg ciprofloxacin, 2 μg clarithromycin, 2 μg clindamycin, 5 μg erythromycin, 10 μg gentamicin, 10 μg linezolid, 5 μg methicillin, 1 unit penicillin, 2 μg rifampicin, 30 μg teicoplanin and 10 μg tetracycline (MAST Diagnostic Ltd).

### Vancomycin susceptibility testing

Vancomycin susceptibility was tested using a previously described method [7].When growth was observed on the vancomycin plates, the minimum inhibitory concentration (MIC) of the isolate was determined by E-test (Cambridge Diagnostic Service Ltd). Vancomycin MIC levels of below 4 mg/L were considered are sensitive and above 4 mg/L was considered as resistant.

### Statistical analysis

Statistical analysis was performed using Z-Test, with the following equation:

KEY: X_1_- Number of positive LBH isolates, X_2_- Number of positive Maltese isolates, N_1_-Total number of LBH isolates, N_2_-Total number of Maltese isolates, P_1_- Population LBH (X_1_/N_1_), P_2_- Population Malta (X_2_/N_2_).

## Results and Discussion

The aim of this study was to determine the MAR profiles for isolates collected from two European study groups, against a panel of fourteen antibiotics and place this information within the context of current prescribing policies for both cohorts. Of the fourteen antibiotics tested, all isolates were completely sensitive to only three antibiotics: linezolid, teicoplanin and vancomycin. This was not surprising since resistance to these antibiotics is currently rare, due to restricted use of this antibiotic. However, resistant strains of MRSA to these antibiotics have been identified in other studies both in the UK and worldwide [8-16].

**Figure 1:**
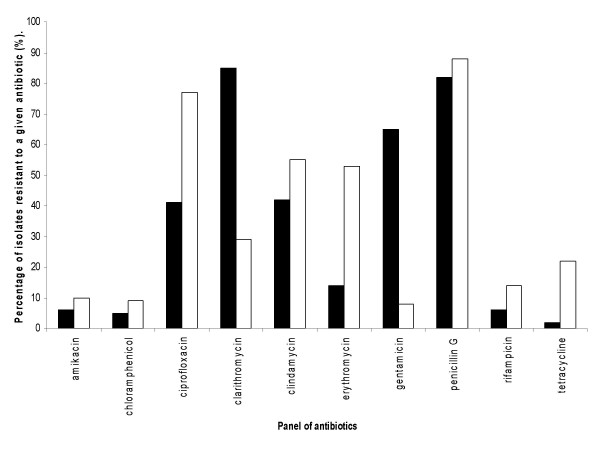
**A Graph illustrating the percentage of methicillin-sensitive *S. aureus *isolates showing resistance to a panel of antibiotics (no resistance was detected for the antibiotics: methicillin, vancomycin, linezolid and tecioplanin)**. MSSA Isolates were collected from LBH (n = 114) and Malta (n = 93). KEY: Black bar represents LBH MSSA isolates and white bars represent Maltese MSSA isolates.

**Figure 2:**
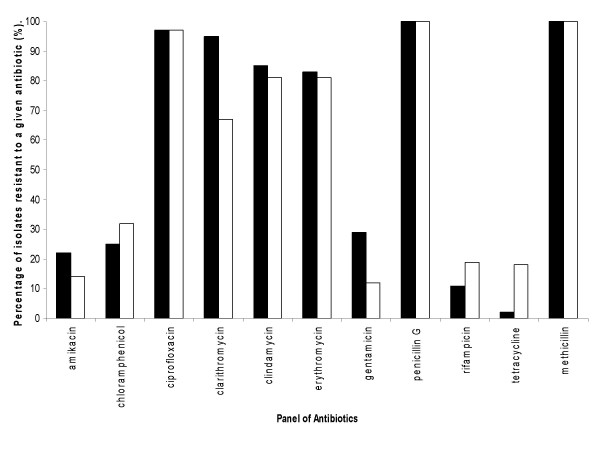
**Graph illustrating the percentage of methicillin-resistant *S. aureus *isolates showing resistance to a panel of antibiotics (no resistance was detected for the antibiotics: vancomycin, linezolid and tecioplanin)**. MRSA isolates were collected from LBH (n = 257) and Malta (n = 216). KEY: Black bar represents LBH MRSA isolates and white bars represent Maltese MRSA isolates.

When comparing the results between MSSA (Figure 1) and MRSA isolates (Figure 2), it could be seen that the MRSA isolates were predominantly resistant to a greater range of antibiotics. Figure 1 shows the resistance level for individual antibiotic results for the MSSA isolates from both isolate groups (LBH and Malta). Of the fourteen antibiotics used the isolates were resistant to ten of the antibiotics and all were sensitive to methicillin; in addition to linezolid, teicoplannin and vancomycin (data not shown in graph). The highest level of resistance was recorded with the antibiotic penicillin G in both isolate cohorts and high levels of resistance (above 70%) were also seen to clarithromycin and ciprofloxacin Low levels of resistance (< 20%) were seen to the antibiotics; amikacin, chloramphenicol, rifampicin and tetracycline in both cohorts, although the level of resistance to tetracycline was slightly higher in the Maltese group (22%).

Statistical analysis showed a significant difference between sensitivity of the MSSA to five of the antibiotics from the LBH and Maltese cohorts (p < 0.05). The LBH isolates showed a higher percentage of resistance to clarithromycin and gentamicin, whereas the Maltese isolates showed a higher percentage of resistance to ciprofloxacin, erythromycin and tetracycline resistance.

Table 1 illustrates the number of MSSA isolates showing resistance a given number of antibiotics of different groups. The isolates show a range of values with some strains showing complete sensitivity to the drugs in the test panel whilst other show resistance to up to eight different antibiotics, with a mean number of resistances of 4 and 3 antibiotics in the LBH and Maltese cohorts respectively.

**Table 1:** The percentage of isolates within a specified cohort showing resistance to a given number of antibiotics (N = 0-10 antibiotics)

Isolates	The number of antimicrobials to which isolates were resistant.										
	0	1	2	3	4	5	6	7	8	9	10
LBH MSSA	0.8	5.1	16.1	28	24.6	19.5	2.5	1.7	1.7	----	----
LBH MRSA	----	----	1.2	1.5	5.4	6.5	36.5	28.1	14.7	4.6	1.5
Maltese MSSA	3.2	7.5	18.3	25.8	22.6	12.9	6.5	2.2	1	----	----
Maltese MRSA	----	----	0.5	5.6	10.6	9.3	29.6	28.2	10.2	4.6	1.4

Figure 2 shows the results for the MRSA isolates from both countries, these results demonstrate very different patterns and levels of resistance to the antimicrobials tested (figure 2) when compared to the MSSA isolates. With reference to figure 2 it can be seen that resistance levels of up to 50% were reported to six of the antibiotics tested in both cohorts. Concentrating on the MRSA isolates from the LBH, it can be seen that resistance levels of greater than 80% were seen to six of the fourteen antibiotics, namely ciprofloxacin, clarithromycin, clindamycin, erythromycin, two of which were β-lactams. The total resistance to the β-lactam antibiotics is unsurprising as all isolates in figure 2 are MRSA and therefore inherently resistant to this class of antibiotic. The high levels of resistance seen to erythromycin, clarithromycin and clindamycin, may in part be due to a single resistance mechanism that affect all of these antibiotics. These three antibiotics share a similar mode of action against the bacterial cell effecting the 50s ribosomal subunit and belong to the; Macrolides (erythromycin, clarithromycin), Lincosamides (clindamycin) and Streptogramins (MLS) class of antibiotic. There are two common mechanisms of resistance against MLS antibiotics, the first encoding by the *erm *gene, leads to the modification of the target ribosome, whereas the second mechanisms of resistance is mediated by two classes of active efflux pumps [17].

The Z-test showed significant differences in the level of resistance to both clarithromycin and gentamicin in the LBH isolates (p < 0.05) when compared to the Maltese MRSA isolates. Interestingly, the Maltese isolates showed increased levels of resistance to tetracycline when compared to the LBH strains (p < 0.05).

The MRSA isolates from both countries were resistant to between 2 and 10 antibiotics, with a mean resistance to six of the antimicrobials in the panel (LBH and Maltese isolates respectively; Table 1).

Previous work carried out by the current authors investigated the strain types within the culture collection by pulse field gel electrophoresis (PFGE). The results of this work showed that the isolates from the two countries had similar DNA profile patterns. The majority of the isolates collected from the UK and Malta were determined to be epidemic MRSA (EMRSA) -15 (53% and 59%; UK and Malta respectively) and -16 (14.3% and 2.1% UK and Malta respectively). A distinct strain was found in the Maltese group which the authors determined to be a local EMRSA particular to Malta and accounted for (14.4%). No correlation of MAR profiles and strain type could be determined [18].

It is proposed that the data presented could contribute to the improvement of current antibiotic prescription policies (APP). In the APP from the LBH, gentamicin is advocated to treat MSSA infection [19]; however, using the data recorded in this study the use of this antibiotic would be limited, due to high levels of resistance (60%). These resistance levels would suggest that in three out of five cases treatment with gentamicin would potentially fail. In Malta the AAP for MSSA infection are clarithromycin and clindamycin [20]; once again using the data recorded in this study the use of clindamycin would be limited as resistance to this antibiotic was demonstrated to be 55%. Furthermore 29% of the MSSA isolates would also be resistance to clarithromycin, interestingly of these isolate 82% (22/27) were resistance to both clarithromycin and clindamycin. This would mean that for almost one quarter of isolates the current treatment would fail. Both in the UK and Malta this could lead to an increase in hospitalisation and progression of infection following treatment failure. These observations highlight the need for a clear understanding of the dynamics of local antibiogram profiles which can then inform local prescribing policy. For isolates that are methicillin-resistant, vancomycin is advocated [19], a similar situation is observed in Maltese hospitals [20]. With the data from this current study, vancomycin as a form of treatment would work in all cases of MRSA investigated in this study. A recent survey examined the antibiotics used in the treatment of MRSA infection in the UK, the findings of this work showed that main treatment consisted of vancomycin singly or in combination [21]. Whilst resistance to this antibiotic remains rare cases have been reported [8,9,11] and therefore increased application may potentially drive the future development of vancomycin resistant strains.

MAR profiles as well as resistance levels to individual antibiotics were determined from the data in this study. This data yielded a large number of different MAR profiles but the frequency of appearance in most cases was extremely low. As a consequence a frequency cut off value was implemented with MAR profiles containing 10 or more isolates being included in the analysis. The MSSA cohort contained 45 (LBH) and 52 (Malta) profiles; a total of nine profiles were present in both countries. The majority of profiles contained less than ten isolates; however, two profiles (one from each cohort) contained ten or more isolates. The profile (gentamicin, penicillin G and clarithromycin) in the LBH cohort contained seventeen isolates (15%, 17/114) and the second profile from the Maltese group (penicillin G and ciprofloxacin) contained ten isolates (11%, 10/93).

Within the MRSA groups a total of 42 MAR profiles were identified in the LBH isolates and 62 profiles in the Maltese strains with 22 profiles being common to both countries. The predominant MAR profiles from both countries can be seen in Table 2, these profiles accounted for 66% (170/256) of the isolates from the LBH and 48% (104/216) of the Maltese isolates. The remaining profiles in both cohorts, containing between one to ten isolates, accounted for 34% (86/256) of the LBH and 52% (112/216) of the Maltese isolates. The most frequent profile within LBH and Maltese MRSA cohorts contained a combination of six and seven antibiotics, with a MAR profile both containing penicillin G, methicillin, ciprofloxacin, clindamycin, clarithromycin and erythromycin (Table 2). A recent study by Comceoção and colleagues [22] examined the MAR profiles from a small hospital in the Azorean islands. This group found two predominant profiles in *circa *86% of MRSA isolates (consisting of profile one; methicillin, penicillin, ciprofloxacin and erythromycin and profile two; methicillin, penicillin and ciprofloxacin). They also reported that all their isolates were sensitive to linezolid, teicoplanin and vancomycin [22], this sensitivity profile was also observed in the isolates in the current study. A second study by Gales and colleagues (2009) determined antimicrobial resistance levels in clinical isolates of MRSA in Brazil [23]. This study reported that these isolates were highly resistant to erythromycin (94%), clindamycin (87.9%) and ciprofloxacin (91.4%). Another study focusing on MRSA isolates collected from a cutaneous infection in a pediatric department demonstrated resistance levels of 70.5% to erythromycin in isolates of MRSA [24]. Despite these studies (including the current work) being carried out in different countries and in varying clinical settings, high levels of resistance to erythromycin, clindamycin and ciprofloxacin were evident in all cases. These particular antibiotics are well used and commonly indicated in treatment policies so increased levels of resistance is not surprising. It is clear that monitoring resistance levels and updating prescribing policies is not only good clinical practice, but also more cost-effective for the hospitals in terms of minimising long hospital stays and the use of antibiotics to which the bacterium may be resistant.

**Table 2:** Illustrates the predominant multiple antibiotic resistant (MAR) profiles identified in the MRSA isolates from the LBH and Malta

Multiple antibiotic-resistant (MAR) profiles	Percentage (%) of isolates showing the indicated resistance pattern.	
	LBH (n = 256)	Malta (n = 216)
AK, G, PG, M, CD, E, Clar, Cip	5.1	-
Ak, PG, M, C, CD, E, Clar, Cip	4.7	-
Ak, PG, M, CD, E, Clar, Cip	4.3	-
G, PG, M, CD, E, Clar, Cip	12.8	-
PG, M, C, CD, E, Clar, Cip	8.2	20.8
PG, M, CD, E, Clar, Cip	31.1	21.8
PG, M, Cip	-	5.6

AK: amikacin; C: chloramphenicol; Cip: ciprofloxacin; Clar: clarithromycin; CD: clindamycin; E: erythromycin; G: genatmicin; M: methicillin and PG: penicillin G.

Inappropriate use of antibiotics is well known, studies carried out in the 1990's suggested that as many as 50% of prescription policies were not fully effective [25,26]. Indeed data from the current study suggests that there might be potential for treatment failure against MSSA infection due to an increase in resistance in both the UK and Maltese isolates.

This may, then lead on to challenges in terms of resistance and selective pressure, Burke (1998) [27] recognised that resistance to antibiotic was easy to promote but difficult to reverse. This can clearly be seen in the rise of MRSA infection in the UK, in 1991 MRSA only accounted for 2% of bacteraemia cases caused by *S. aureus*, however by 2000, MRSA represented 42% of *S. aureus *isolates from bacteraemia cases [2]. It is encouraging to note that this level of resistance has decreased from 2000, to 32% in 2008 [5]. Therefore the question remains: do we need to revaluate the way the proscription policies are determined locally and nationally?

Cooke and Holmes [28] proposed there need to be better antibiotic stewardship, in the possible form of "antibiotic care bundle" (ACB) [28]. These care bundles aim to select antibiotics that are most likely to present a fully therapeutic solution, whilst reducing both potential side effects and the risk of developing resistance to the antibiotic in use. Some of the key components suggested by the authors indicate that the bundle should be adapted for local needs and the policy should have input from local microbiologists and pharmacists. However the authors did not define what they determined to be local. It may be speculated that within one hospital, different wards or clinical areas may require their only ACB. The authors also suggest that the prescribing policy should be monitored and altered when and if required, following the apparent development of resistance to one or more antibiotics [28].

Antibiotic care bundles has been implemented in Scotland since 2005 and are run by a multidisciplinary antimicrobial management team (AMT) consists of; lead doctor, pharmacist, microbiologist and a representative of senior management [29]. Two recent reports from the USA have demonstrated the potential of ACB in reducing antibiotic resistance in addition to show the cost effectiveness and potential saving on drug treatment [30,31]. Drew (2009) [30] although does highlight the possible difficulty in implementing ACB such as; the need of appropriate personnel to run the project, initial funding of the project both financially and the time that needs to be assigned to the project, as well as potentially resistant to the ACB from colleagues. However on balance the finding of these studies show the potential benefit of ACB out weight the initial problem, but understanding the local microbial antibiotic resistance pattern within a hospital would be required before any care bundle could be developed and implemented.

## Conclusion

In conclusion, this report shows that two study groups geographically separated by large distances have similar antibiotic resistance profiles in their hospital isolates of *S. aureus*, despite differences in antibiotic treatment regimens indicated in the respective hospital policies. The importance of monitoring the level of not only methicillin resistance but also the resistance levels of other antibiotics is also clearly demonstrated. Currently in the UK, the government has introduced a mandatory reporting scheme for methicillin resistance in *S. aureus*. Due to the high level of resistance recorded to some of the antibiotics used in this study, it could be suggested that this type of scheme needs to be extended to include other antibiotics where there is the potential for resistance level to reach 100%. The data presented in this paper suggests that the continued monitoring of local antibiotic resistance profiles is essential. The resultant epidemiology is important in informing local prescribing policies and the early identification of emerging resistance patterns, allowing for the production of responsive therapy regimens in response to ever changing microbial challenges.

## Competing interests

The authors declare that they have no competing interests.

## Authors' contributions

SG carried out the analysis of samples, performed the statistical analysis and drafted the manuscript. PC organised the coordination of sample collection in Malta. JR and ACH both undertook some of the MAR analysis. SE participated in its design and coordination. MDF devised the study, participated in its design and coordination. All authors have read and approved the final manuscript.
